# Assessment of Administration and Receipt of COVID-19 Vaccines by Race and Ethnicity in US Federally Qualified Health Centers

**DOI:** 10.1001/jamanetworkopen.2021.42698

**Published:** 2022-01-10

**Authors:** Megan B. Cole, Julia R. Raifman, Sabrina A. Assoumou, June-Ho Kim

**Affiliations:** 1 Department of Health Law, Policy, and Management, Boston University School of Public Health, Boston, Massachusetts; 2 Section of Infectious Diseases, Department of Medicine, Boston University School of Medicine, Boston, Massachusetts; 3 Section of Infectious Diseases, Department of Medicine, Boston Medical Center, Boston, Massachusetts; 4 Ariadne Labs, Brigham and Women’s Hospital, Harvard T.H. Chan School of Public Health, Boston, Massachusetts; 5 Division of General Internal Medicine & Primary Care, Brigham and Women’s Hospital, Boston, Massachusetts

## Abstract

This cohort study investigates the dispensation of the COVID-19 vaccination at federally qualified health centers in the US and whether the vaccine has been distributed equitably to people of different races and ethnicities.

## Introduction

Federally qualified health centers (FQHCs) care for low-income, racially and ethnically diverse, medically underserved populations disproportionately affected by COVID-19 and its associated health inequities.^1^ As trusted, accessible entities, FQHCs may mitigate further inequities by providing access to COVID-19 vaccination in communities most affected by COVID-19 that have often been least likely to have access to vaccines.^2^ The objectives of this study were to examine (1) COVID-19 vaccination administration rates at US FQHCs by race and ethnicity and (2) the racial and ethnic equity in vaccine receipt at FQHCs.

## Methods

We conducted a retrospective cohort study using biweekly Health Resources & Services Administration Health Center COVID-19 Survey data from January 8 to July 2, 2021,^3^ of all FQHCs in the US. The data reported counts of vaccinations per FQHC by race and ethnicity with an average biweekly response rate of 81%. Patients self-identified as Hispanic, non-Hispanic American Indian and Alaska Native, non-Hispanic Asian, non-Hispanic Black, and non-Hispanic White (hereafter, American Indian and Alaska Native, Asian, Black, and White) individuals, or as individuals of other race (the other category includes the following races and ethnicities or multiple races: Hawaiian, Other Pacific Islander, and multiracial). The secondary data source was the 2019 Uniform Data System, which includes annual FQHC-level data reported by all FQHCs that capture information on patient, organizational, and performance characteristics of the FQHCs. We excluded FQHCs reporting data for less than 80% of all biweeks (n = 234), those located in US territories (n = 32), and those with fewer than 10 vaccinations per week (n = 22). Boston University’s Institutional Review Board deemed the study exempt and waived the requirement for informed consent because data were publicly available. This study followed the Strengthening the Reporting of Observational Studies in Epidemiology (STROBE) guideline.

The unit of analysis was the FQHC biweek. The outcomes of interest were (1) the cumulative percentage of initiated vaccines administered at FQHCs according to the recipient’s self-reported race or ethnicity and (2) the vaccination equity ratio of the number of observed cumulative, initiated vaccinations within a racial or ethnic group divided by the number of expected vaccinations within the group, where the expected number is based on the percentage of the FQHC population that belongs to that racial or ethnic group. We used linear probability models to estimate the association between calendar time (biweeks) and the 2 outcome measures, which were estimated separately for each self-reported racial group. All models applied population-size denominator weights and produced robust SEs clustered at the FQHC level.

*P* values were 2-tailed, and statistical significance was set at α = .05. Analyses were performed using Stata, version 17.0 (StataCorp, LLC).

## Results

**Figure 1.**
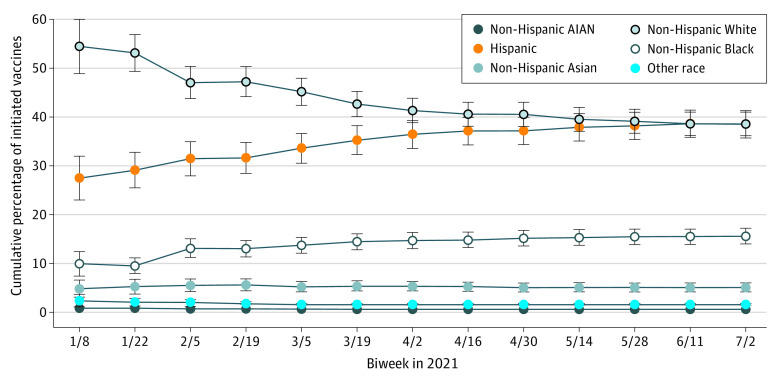
Cumulative Percentage of Initiated COVID-19 Vaccines Administered at US FQHCs by Race or Ethnicity From January to July 2021 The race and ethnicity of 81.3% of all vaccination recipients was known; race and ethnicity information for 18.7% of vaccination recipients was unknown and was thus excluded from the denominator. All racial or ethnic subgroups are mutually exclusive and collectively exhaustive and include 30 852 American Indian or Alaska Native (AIAN), 618 024 Asian, 684 792 Black, 2 181 502 Hispanic, 1 974 800 non-Hispanic White individuals, and 116 683 individuals of other race (including multiple races, Hawaiian, and other Pacific Islander). Each biweek reflects the cumulative total number of vaccinations administered as of the end of the 14-day biweek. In any given biweek, all percentages across racial or ethnic groups add up to 100%. FQHCs indicates federally qualified health centers.

Among 1096 FQHCs in the study sample that served 25.9 million patients (14.6 million [56%] female and 11.3 million [44%] male individuals; 181 090 [0.7%] American Indian and Alaska Native, 750 230 [2.9%] Asian, 4.5 million [17.5%] Black, 10.9 million [42.0%] Hispanic, and 9.0 million [34.9%] White, and 517 400 [2.0%] other race individuals), 5 606 679 initial vaccinations were administered to patients with a known race or ethnicity (race and ethnicity were unknown in 18.7% of patients who received COVID-19 vaccines) during the study period. The cumulative proportion of patients who received vaccines at FQHCs and self-identified as Black or Hispanic increased over time (Figure 1). By July 2, 2021, 61.4% of all cumulative, initiated vaccines administered at FQHCs were to patients who self-identified as American Indian or Alaska Native (30 852), Asian (618 024), Black (684 792), Hispanic (2 181 502), or other race and ethnicity (116 683; other included the following races and ethnicities or multiple races: Hawaiian, Other Pacific Islander, and multiracial). Of this proportion, less than 1% of vaccines were administered to American Indian or Alaska Native patients, 5.1% in Asian patients, 15.6% in Black patients, 38.5% in Hispanic patients, and 1.5% in patients of other or multiple races.

**Figure 2.**
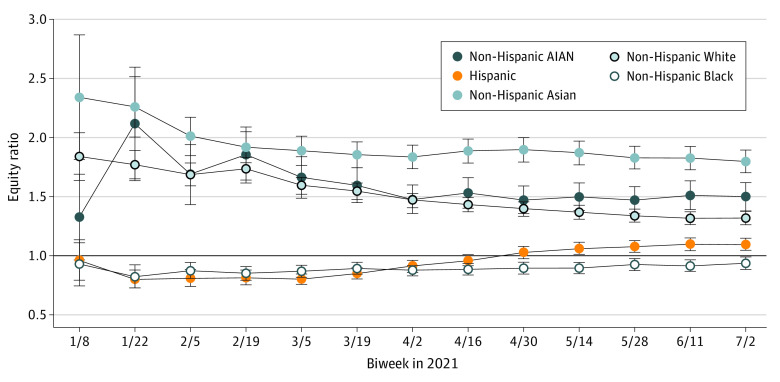
COVID-19 Vaccine Equity Ratios for Cumulative Initiated Vaccinations at US FQHCs From January to July 2021 An equity ratio of less than 1.0 suggests that the racial or ethnic group received fewer vaccinations than it would have received if vaccinations were equitably administered by race and ethnicity. An equity ratio of greater than 1.0 suggests that the racial or ethnic group received proportionally more vaccinations than it would have received if vaccinations were equitably administered by race and ethnicity. The 95% CIs that cross 1.0 or exceed 1.0 indicate equity or excess equity. To calculate equity ratios, racial and ethnic composition of each federally qualified health center (FQHC) was based on 2019 Uniform Data System data, where on average, patients at FQHCs self-identified as American Indian or Alaska Native (AIAN; 0.7%), non-Hispanic Asian (2.9%), non-Hispanic Black (17.5%), Hispanic (42.0%), non-Hispanic White (34.9%), and other race (2.0%; other included multiple races, Hawaiian, and other Pacific Islander). Each biweek reflects the cumulative total number of vaccinations administered as of the end of the 14-day biweek.

When accounting for the racial and ethnic composition of FQHC populations, American Indian or Alaska Native, Asian, and non-Hispanic White patients were initially more likely to receive the vaccine, whereas Black and race Hispanic patients were less likely to receive the vaccine (Figure 2). However, equity changed over time, and by April 16, 2021, all racial and ethnic groups experienced statistical equity (ratio ≥1.0) except for Black patients, who, by July 2, 2021, had an equity ratio of 0.94 (95% CI, 0.88-0.99).

## Discussion

Federally qualified health centers have provided critical access to COVID-19 vaccinations for patients from diverse racial and ethnic groups. As of July 2021, FQHCs administered 61.4% of their vaccines to patients of races and ethnicities other than White compared with 40% administered to racial and ethnic minority groups in the general US population.^4^ Vaccine administration at FQHCs was equitable for American Indian or Alaska Native, Asian, and Hispanic populations, but there were inequities for the Black population, although these inequities were smaller compared with those of the general US population.^2^ Initial inequities in vaccine administration in the Hispanic population were likely driven by state-dictated, age-based vaccine eligibility.

The present study suggests that governments should continue to sustainably fund and prioritize the use of FQHCs as major vaccine administration sites to improve vaccination equity through the Health Center COVID-19 Vaccine Program.^5^ This includes direct allocation of vaccines; use of FQHC-based mobile clinics, pop-up vaccination sites, and school-based sites; and targeted outreach for Black and Hispanic patients, including pediatric populations.^6^ Study limitations include the use of facility-level data without individual-level characteristics, such as age. In addition, equity ratios are sensitive to measurement error in smaller racial and ethnic populations, and missing racial and ethnic data may bias findings. Nonetheless, as policy makers work toward increasing COVID-19 vaccine equity, they must invest in and learn from FQHCs by meeting patients where they are geographically and continuing outreach by trusted community clinicians.
